# Coexisting Chronic Rhino-Cerebral Mucormycosis and Actinomyces Infection: A Case Report and Review of the Literature

**DOI:** 10.7759/cureus.59694

**Published:** 2024-05-05

**Authors:** Victor D Acuña-Rocha, José A Ramírez-Vázquez, Luis A González Torres, Jenny C López-Zamarrón, Luz Del Cármen Tarín-Arzaga

**Affiliations:** 1 Internal Medicine, Hospital Universitario José Eleuterio González, Universidad Autónoma de Nuevo León, Monterrey, MEX; 2 Gastroenterology and Digestive Endoscopy, Hospital Universitario José Eleuterio González, Universidad Autónoma de Nuevo León, Monterrey, MEX; 3 Hematology, Hospital Universitario José Eleuterio González, Universidad Autónoma de Nuevo León, Monterrey, MEX

**Keywords:** actinomycetes co-infection, actinomyces infection, angioinvasive mucormycosis, rhino-orbito-cerebral mucormycosis, rhinocerebral mucormycosis

## Abstract

Fungal rhino-orbital-cerebral infections present significant treatment challenges, especially in immunocompromised individuals, such as those with diabetes. These infections seldom occur with bacterial co-infections, which complicate their management. This report presents the case of a 74-year-old diabetic male with a long-standing history of left malar pain who experienced rhinorrhea, nasal congestion, and confusion. Diagnostic imaging revealed angioinvasive fungal sinusitis, ultimately attributed to chronic mucormycosis (CM) with concurrent *Actinomyces *infection, a rarely reported occurrence. We employed a comprehensive treatment strategy, which resulted in a successful recovery after 24 days. Although CM is rare, accounting for approximately 5.6% of cases with mucormycosis, it requires thorough diagnostic evaluation and prolonged treatment. The rarity of co-infections like the one we describe underscores the need for an integrated management approach. Histopathological analysis serves as the gold standard for diagnosis, with treatment typically involving surgical and extensive antifungal interventions.

## Introduction

*Mucoraceae* are a family of fungi of the order *Mucorales*. The pathogenic organisms in this order include the *Rhizopus*,* Mucor*,and *Absidia *sp., opportunistic and invasive filamentous fungi that tend to affect rhino cerebral tissues. An aggressive clinical course characterizes it. Fungal sinus infections are categorized into acute fulminant, chronic invasive, noninvasive colonization, and allergic. The invasive subtype is subclassified into granulomatous, acute fulminant, and chronic invasive subtypes. People with diabetes and hematologic malignancies have impaired phagocytic activity. Fungal pathogens take advantage of this situation and invade the host, causing invasive illnesses [1,2]. Immunocompetent host infection occurs in approximately 4% of affected individuals [3]. Chronic mucormycosis (CM) is rare and represents roughly 5.6% of all mucormycotic infections. There is no exact time definition for CM. Gutierrez et al. mentioned that CM describes cases that last more than four weeks [2]. Causative agents can be identified from samples of the affected tissue in 11% to 21.7% of the cases [2,4]. Typical clinical presentations involve headaches, eye affection, nasal cavities, osteomyelitis, and sinuses [5]. Bacterial and viral co-infections with *Mucoraceae *are rare and even rarer with *Actinomyces*. We present a unique CM case in a patient with diabetes type 2 complicated by a co-infection with *Actinomyces* species. Despite several days of hospitalization and interventions, the patient experienced favorable outcomes.

## Case presentation

**Figure 1:**
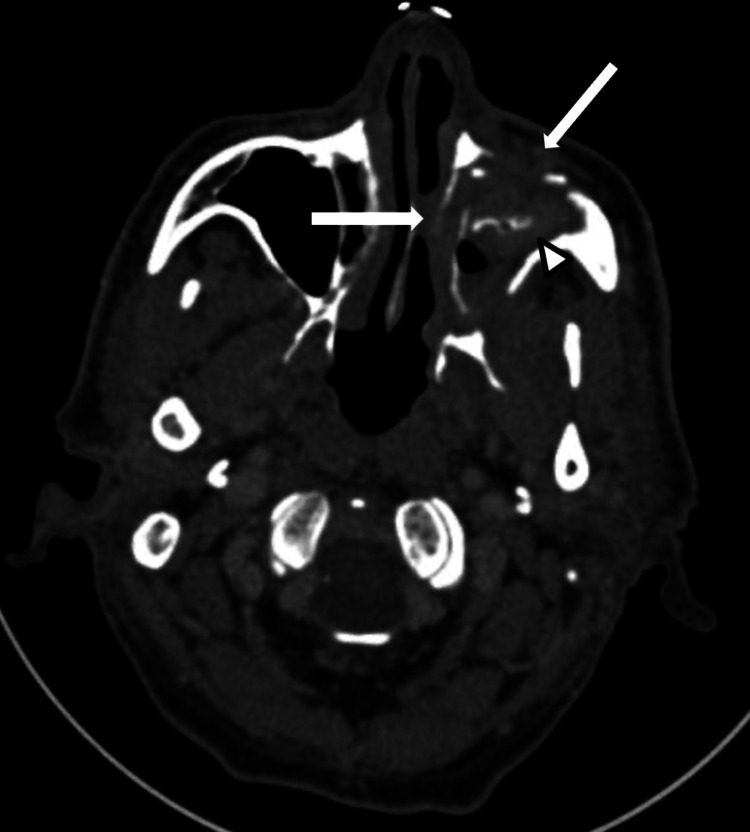
Contrast-enhanced computed tomography (CT) scan of the head and neck. The white arrows indicate the fungal infiltration of the anterior and lateral walls of the left maxillary sinus. The white arrowhead shows the occupation of the left maxillary sinus by heterogeneous material.

A 74-year-old male farmer with a previous lifetime exposure to biomass smoke and inactive alcoholism, history of well-controlled type 2 diabetes, hypertension, Parkinson's disease (treated with biperiden), benign Hyperplasic prostate (treated with transurethral prostatectomy six years ago), and history of right pulmonary lobectomy secondary to traumatic injury, presented with a five-year history of moderate-severe left malar pain. He reported a lack of adherence to his medication regime. Four months before his visit, he started with rhinorrhea, nasal congestion, halitosis, and cacosmia, experienced a single episode of abundant epistaxis, and, two months later, intermittent episodes of confusion that required hospitalization. Computed tomography (CT) scan of the head and neck (Figure 1) suggested a sinus infection.

**Figure 2:**
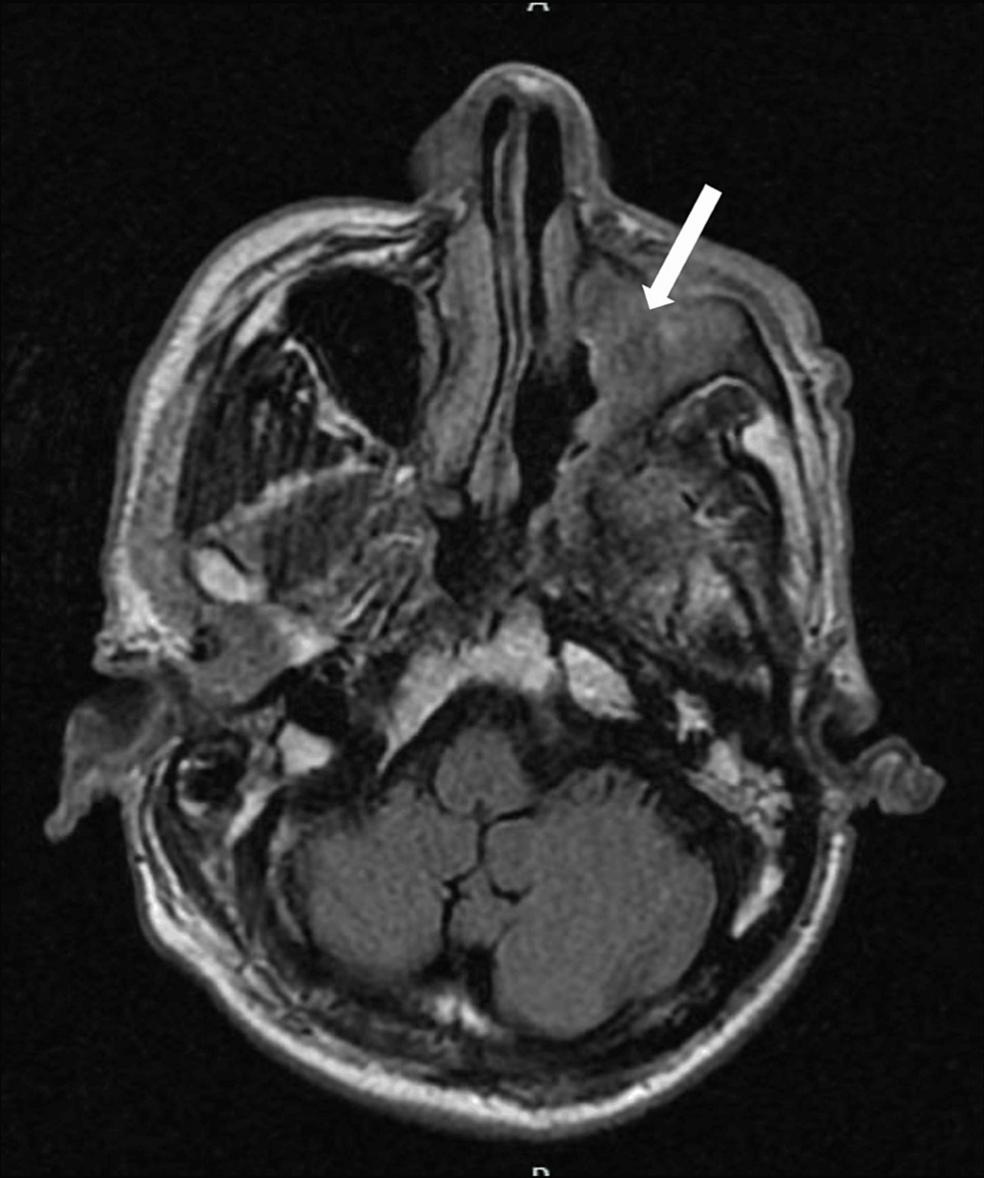
T2-Flair gadolinium-enhanced magnetic resonance imaging (MRI) scan of the head and neck. The white arrow shows the occupation of the left maxillary sinus by heterogeneous material that isointense to the rest of the mucosa.

**Figure 3:**
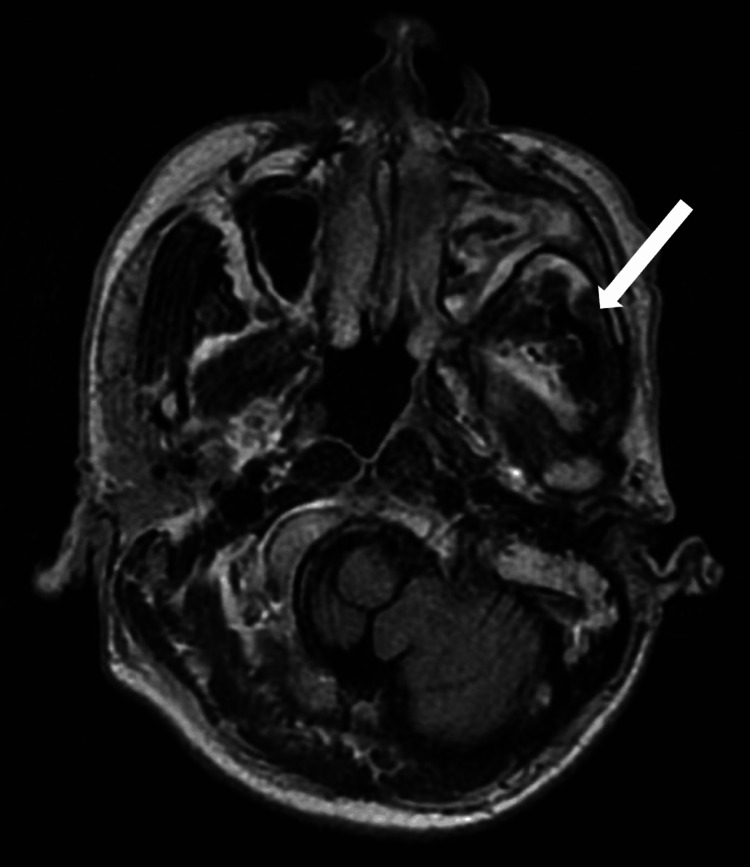
T2-Flair gadolinium-enhanced magnetic resonance imaging (MRI) scan of the head and neck. The white arrow indicates that changes in signal intensity of the masseter space compared to the contralateral side suggest fungal infiltration.

Consecutively, a biopsy of the left middle turbinate and resection of the left maxillary tumor revealed acute and chronic inflammatory processes with tissue necrosis associated with abundant mycotic structures morphologically compatible with Zygomycetes. He arrived for his follow-up visit with biopsy results, after which he was referred to the hospital for an urgent assessment. At the initial evaluation, he presented with normal vital signs, and the physical examination revealed pain upon palpation of the left malar region. Laboratory tests showed 250 mg/dL serum glucose, 1.2 mg/dL creatinine, and normocytic normochromic anemia (Hb 8.54 g/dL). T2-Flair gadolinium-enhanced magnetic resonance imaging (MRI) scan of the head and neck demonstrated erosion of the orbit floor, anterior, medial, and posterior walls, and the maxilla's alveolar and palatine processes (Figures 2, 3).

**Figure 4:**
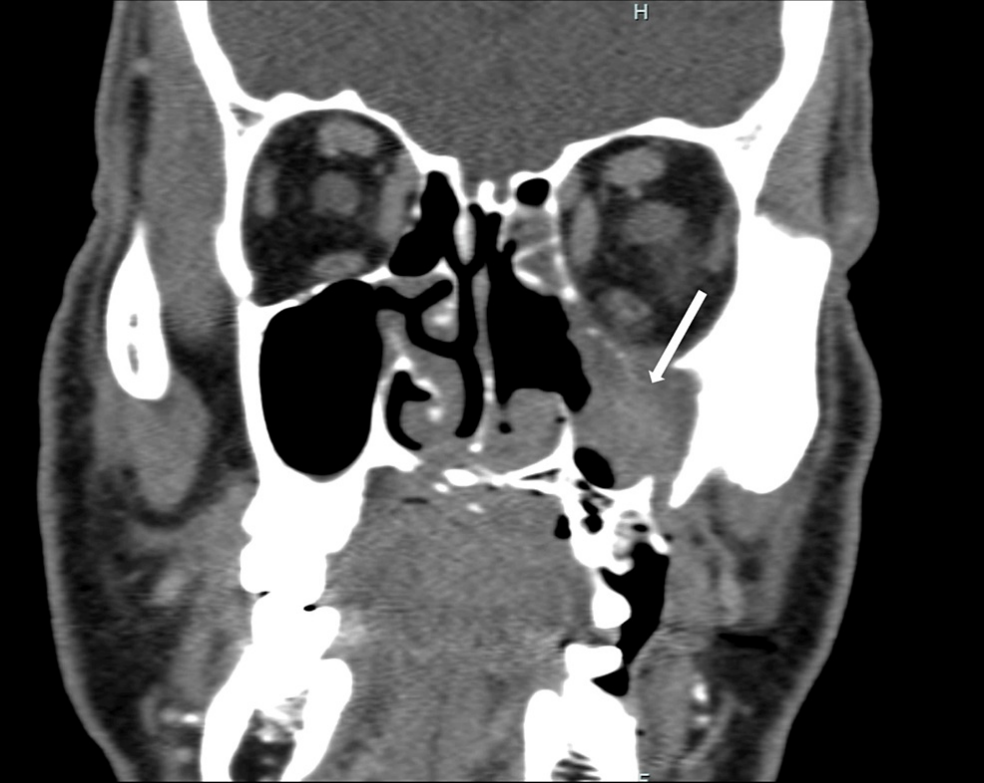
Contrast-enhanced computed tomography (CT) scan of the head and neck. The white arrow indicates the fungal infiltration of the left extraconal space.

The palatine bone and ipsilateral pterygoid processes showed involvement, extending into the surrounding soft tissues of the inferior extraconal space, masseter muscle, and the left parapharyngeal space. Additionally, a contrast-enhanced CT scan (Figure 4) of the head and neck displayed a distinct mass effect on the lateral wall of the nasopharynx. These findings were strongly suggestive of angioinvasive fungal sinusitis. Therefore, we initiated treatment with liposomal amphotericin at 5 mg/kg per day.

We observed substantial clinical improvement by the fourth day of treatment, but the rhinorrhea and halitosis persisted. After the 14th day of hospitalization, the otorhinolaryngology service performed a nasendoscopy. They removed and biopsied the nasal mucosa and the palatine, left concha nasalis inferior, and left maxillary bones. During his stay, the patient presented a hospital-related urinary infection by *Klebsiella* species with extended-spectrum beta-lactamase (ESBL) resistance, which was treated with meropenem TID. Pathology reports revealed the presence of a chronic zygomycete infection with positive culture for non-resistant *Actinomyces* species. After 24 days of admission and full clinical recovery, we discharged him. A month later, he reported a good adherence to the azole treatment and a substantial improvement in his symptoms.

## Discussion

This report is relevant because it describes a rare case of CM with *Actinomyces *sp. infection. We aimed to highlight our diagnostic process and treatment strategies to guide clinicians encountering a similar scenario. Considering the patients’ Immunological status, particularly in patients with otherwise well-controlled diabetes despite adequate glycemic control, one of the limitations of our study is the absence of histopathological image confirmation of the infection due to hospital-patient confidentiality.

As mentioned above, CM is an invasive fungal infection, constituting 5.6% of all mucormycotic infections. Patients with diabetes and hematologic malignancies are immunosuppressed and suffer from impaired phagocytic activity; this can result in opportunistic infections by fungal pathogens. An acid and glucose-enriched environment promotes fungal growth, tissue entry, angioinvasion, thrombosis with necrosis, ischemia, and extension to surrounding tissues [1,2]. Hyperglycemia decreases the secretion of inflammatory cytokines, impairs T-cell function and mobilization/activation of neutrophils, and depresses humoral immunity. Stress-induced hyperglycemia (SIH) is a transient hyperglycemia associated with clinical illness independent of diabetes that may worsen the glycemic status of the patient with type 2 diabetes. SIH facilitates catecholamine release, resulting in an impairment in immunity [6]. Hyperglycemia poses a significant risk to the immune system irrespective of diabetes status.

Gutierrez et al. described the most extensive CM case review, listing 22 reported cases between 1964 and 2014 and reporting a prevalence of 26% in immunocompetent patients [2]. Our patient presented with a history of well-controlled diabetes mellitus, which potentially implies a healthier immune system compared to patients with poorly controlled diabetes. Other cases reported CM infections in a person with diabetes, an 84-year-old person, and an immunocompetent patient [5,7,8]. Mucormycosis in immunocompetent patients needs a more thorough diagnostic workup.

Mucormycosis can be divided into six types based on anatomic location: rhino cerebral, pulmonary, cutaneous, gastrointestinal, disseminated, and uncommon presentations (endocarditis, osteomyelitis, peritonitis, and pyelonephritis) [9]. The most frequent clinical presentation for mucormycosis includes ptosis, ophthalmoplegia, vision loss, and pain in the retrobulbar region, as observed in our patient [2,4,7,8]. The diagnosis of CM mandates microscopic observation of the fungus within the specific tissue. Characteristic histopathological findings are non-septated 90° branching invasive hyphae detectable via hematoxylin-eosin, PAS (periodic acid-Schiff), or Grocott-Gromori staining and, in rare cases, granulomas [2,10]. Co-infections may occur along with *Mucoraceae *sp. [7,10,11]. One case report described a patient with *Actinomyces* co-infection with acute mucormycosis; other reported cases described concomitant pulmonary affection with SARS-CoV-2 and *Aspergillus *infection. *Actinomyces* infection rarely involves the cervicofacial region and usually presents in immunocompetent individuals; our patient presented a concomitant infection of CM and *Actinomyces*.

Concomitant *Actinomyces* and CM differential diagnoses include bacterial infections, infiltrative infections, sinonasal neoplasms, autoimmune disease (Graves), or vascular complications (cavernous sinus thrombosis) [2,5].

As described previously, Gutierrez et al. published the most extensive review on CM cases; they noted that most chronic patients have better survival rates than acute ones (83% vs. 10-35%) and recommend surgical and antibiotic therapy [2]. Most reports have used amphotericin B and azole therapy [2,4,7,8,12]. We administered a six-week course of amphotericin B followed by azole therapy until subsequent follow-up and complete resolution. Actinomyces treatment consisted of a six-week trial of carbapenem therapy considering the concomitance of urinary tract *Klebsiella* ESBL infection. In the case reports we reviewed, antibiotic therapy depended on antibiotic susceptibility; Rani et al. did not comment on the treatment used for the *Actinomyces* co-infection [7]. In both pulmonary cases of co-infection of *Actinomyces* and mucormycosis, antibiogram susceptibilities directed the specific antimicrobial therapy; Fujisaki et al. used ampicillin, while Lin et al. administered piperacillin-tazobactam [13,14].

## Conclusions

In conclusion, our study reveals insights into the complex interplay between immune status and fungal infections, particularly in cases of chronic mucormycosis (CM), which are typically associated with immunocompromised patients. Furthermore, the rarity of bacterial-fungal co-infections emphasizes the importance of thorough evaluation in diagnosing fungal infections. The absence of *Actinomyces* co-infection in CM cases suggests a unique clinical presentation that warrants further investigation.

Our experience underscores the significance of a multidisciplinary approach to patient care, focusing on survival and quality of life. Additional research and comprehensive diagnostic strategies are necessary to fully understand and manage these complex clinical scenarios.

Our study contributes valuable insights into fungal infection management, emphasizing the need for tailored approaches based on immune status and thorough evaluation. By addressing these critical factors, we aim to enhance patient outcomes and advance clinical practice in this area.
